# Transcriptomic comparison of seeds and silique walls from two rapeseed genotypes with contrasting seed oil content

**DOI:** 10.3389/fpls.2022.1082466

**Published:** 2023-01-13

**Authors:** Xupeng Guo, Na Yan, Linpo Liu, Xiangzhen Yin, Yuhong Chen, Yanfeng Zhang, Jingqiao Wang, Guozhi Cao, Chengming Fan, Zanmin Hu

**Affiliations:** ^1^ State Key Laboratory of Plant Cell and Chromosome Engineering, Institute of Genetics and Developmental Biology, Innovation Academy for Seed Design, Chinese Academy of Sciences, Beijing, China; ^2^ Hybrid Rapeseed Research Center of Shaanxi Province, Yangling, Shaanxi, China; ^3^ Institute of Economical Crops, Yunnan Agricultural Academy, Kunming, Yunnan, China; ^4^ College of Advanced Agricultural Sciences, University of Chinese Academy of Sciences, Beijing, China

**Keywords:** photosynthesis, seed oil content, silique development, gene coexpression, molecular regulation network

## Abstract

Silique walls play pivotal roles in contributing photoassimilates and nutrients to fuel seed growth. However, the interaction between seeds and silique walls impacting oil biosynthesis is not clear during silique development. Changes in sugar, fatty acid and gene expression during *Brassica napus* silique development of L192 with high oil content and A260 with low oil content were investigated to identify key factors affecting difference of their seed oil content. During the silique development, silique walls contained more hexose and less sucrose than seeds, and glucose and fructose contents in seeds and silique walls of L192 were higher than that of A260 at 15 DAF, and sucrose content in the silique walls of L192 were lower than that of A260 at three time points. Genes related to fatty acid biosynthesis were activated over time, and differences on fatty acid content between the two genotypes occurred after 25 DAF. Genes related to photosynthesis expressed more highly in silique walls than in contemporaneous seeds, and were inhibited over time. Gene set enrichment analysis suggested photosynthesis were activated in L192 at 25 and 35 DAF in silique walls and at both 15 and 35 DAF in the seed. Expressions of sugar transporter genes in L192 was higher than that in A260, especially at 35 DAF. Expressions of genes related to fatty acid biosynthesis, such as *BCCP2*s, *bZIP67* and *LEC1*s were higher in L192 than in A260, especially at 35 DAF. Meanwhile, genes related to oil body proteins were expressed at much lower levels in L192 than in A260. According to the WGCNA results, hub modules, such as ME.turquoise relative to photosynthesis, ME.green relative to embryo development and ME.yellow relative to lipid biosynthesis, were identified and synergistically regulated seed development and oil accumulation. Our results are helpful for understanding the mechanism of oil accumulation of seeds in oilseed rape for seed oil content improvement.

## Introduction

Light is one of the most important environmental factors not only for plant vegetative growth and development but also for seed development and storage reserves. During embryogenesis, young seeds of many plant species, such as rapeseed (*Brassica napus*) and soybean (*Glycine max*), are green and can carry out photosynthesis at a gradually increasing rate until the onset of desiccation, although light reaching the seed is low due to the encapsulation of silique walls, which are photosynthetically active (Eastmond et al., 1996; Goffman et al., 2005; Allen et al., 2009). Furthermore, light markedly influences chloroplast biogenesis in green seeds (Liu et al., 2017), which contain chloroplasts with thylakoid structures and enzymes of typical photosynthetic machinery, although they are predominantly sink tissues (Puthur et al., 2013). Photosynthesis of the green seed is of biofunctional significance for seed development, such as by providing O_2_, ATP and reductants to control biosynthetic fluxes by improving the energy supply (Willms et al., 1999; Borisjuk et al., 2004; Borisjuk et al., 2005; Goffman et al., 2005; Rolletschek et al., 2005), improving the efficiency of carbon storage through RubisCO (ribulose 1,5-bisphosphate carboxylase/oxygenase) acting without the Calvin cycle (King et al., 1998; Schwender et al., 2004a; Goffman et al., 2005) and inducing deposits of storage reserves during embryo maturation (Borisjuk et al., 2004; Liu et al., 2017; Tan et al., 2020).

Seed development is the growth of maternal and filial tissues in higher plants, beginning with a double-fertilization process and ending with a dormant seed, and consists of embryogenesis, maturation and desiccation (Borisjuk et al., 2004; Le et al., 2010). During seed development, the plant seed receives photosynthetically assimilated carbon, mostly in the form of sucrose, from the mother plant for the synthesis of main storage products such as oils, starch and proteins. The seed size and accumulation of seed storage reserves are crucial to advancing crop yield and are controlled by different seed stages. Each stage of seed development is characterized by specific sequences of physiological traits, molecular events and metabolic profiles. Embryogenesis features extensive cell division, orchestrated differentiation and expansion, and embryo maturation is characterized by the accumulation of seed storage reserves in the highly specialized storage tissues, embryo and endosperm (Borisjuk et al., 2004; Baud et al., 2008; Le et al., 2010; Wendrich and Weijers, 2013). Metabolically, a high glucose level maintains the capacity of cells to divide in early seed development, whereas a certain sucrose level represents a signal for differentiation and triggers the seed maturation process (Wobus and Weber, 1999; Hill et al., 2003; Borisjuk et al., 2004; Morley-Smith et al., 2008; Aguirre et al., 2018). Genetically, four major conservative regulators, LEAFY COTYLEDON1 (LEC1), ABSCISIC ACID INSENSITIVE3 (ABI3), FUSCA3 (FUS3) and LEAFY COTYLEDON2 (LEC2), interact to form different complexes that regulate different aspects of seed development from early to late embryogenesis and accumulation of storage compounds in dicots and monocots (Fatihi et al., 2016; Boulard et al., 2017; Lepiniec et al., 2018; Tian et al., 2020).

Seed oil content (SOC) is an important and complex agronomic trait for oilseed crops and is regulated by multiple factors. Triacylglycerols (TAGs) are the major constituent of plant storage oils; their biosynthesis comprises two main biological processes, fatty acid biosynthesis mainly in chloroplasts and assembly of acyl chains into TAGs in the endoplasmic reticulum that are finally stored in the oil body. Furthermore, more than 60% of the total carbon flux is directed into storage TAGs in the seed (approximately 45% of dry weight) in oilseeds such as *B. napus* (Schwender et al., 2004a; Schwender et al., 2004b). The primary substrate for seed oil synthesis is sucrose imported by long-distance transport from the photosynthetic tissues that is metabolized to generate acetyl-CoA units. Then, sucrose is mainly from maternal tissues, and the silique wall in *B. napus* and photosynthetic activities in silique walls are closely correlated with SOC (Baud and Lepiniec, 2010; Hua et al., 2012; Tan et al., 2015; Zhu et al., 2018; Li et al., 2019; Shahid et al., 2019). In addition to the carbon skeleton supply through photosynthesis, light, as a molecular signal, also modulates oil biosynthesis by affecting fatty acid biosynthesis. The first key rate-limiting enzyme, heteromeric acetyl-CoA carboxylase (ACCase), catalyses the first committed step of *de novo* fatty acid synthesis in chloroplasts. Light directly modulates a regulatory site of the plastidic prokaryotic form of ACCase *via* a signal transduction pathway of a redox cascade and indirectly modulates its catalytic activity *via* stromal pH and Mg^2+^ concentration (Nakamura and Yamada, 1979; Sasaki et al., 1997; Ye et al., 2020a), and biotin/lipoyl attachment domain containing (BADC) and biotin carboxyl carrier protein (BCCP, a subunit of ACCase) can act as pH sensors required for light-dependent switching of ACCase activity (Ye et al., 2020a). Light also enhances the interaction between carboxyltransferase interactors (CTIs) and alpha-carboxyltransferase (α-CT, a subunit of ACCase), which in turn attenuates carbon flux into FAS (Ye et al., 2020b). In addition, light can provide stoichiometric amounts of ATP, NADPH, and NADH for each sequential addition of an acetyl unit to the growing chain of the fatty acid through photosynthesis (Puthur et al., 2013).

Overall, light has important functions for seed growth and development in providing enough carbohydrates from the silique wall and O_2_ and ATP from the green seed through photosynthesis. However, the interaction between seeds and silique walls impacting oil biosynthesis is not clear during silique development. In this study, developing seeds and silique walls of two *B. napus* genotypes, L192 and A260, with different SOCs were compared regarding the contents of sugar and fatty acids and transcriptomic changes to explain the relationships between photosynthesis of the silique wall and seed oil content. Through WGCNA, we found that important biological pathways, such as photosynthesis, fatty acid biosynthesis and oil storage, were controlled by several modules during the development of siliques.

## Materials and methods

### Rapeseed planting and sampling

The agronomic traits of two winter-type inbred lines of *Brassica nupus*, L192 (high-oil, approximately 55%) and A260 (low-oil, approximately 37%), were stable throughout years of investigation in Yangling, Shanxi Province, China. Under the same field conditions, oleic acid (C18:1, OA), linoleic acid (C18:2, LA), linolenic acid (C18:3, ALA), erucic acid (C22:1, EA), glucosinolate (GLs) and saturated fatty acid (SFA) of mature seeds were measured through near-infrared reflectance spectroscopy (NIRS, Foss NIRSystems Inc., USA), and the seed oil content was measured through nuclear magnetic resonance (NMR, mq-20, Bruker, Germany). Traits of mature dry seeds from L192 and A260 (**Supplementary Table 1**) were confirmed using NIRS and NMR in Yangling in 2016.

The seed or silique wall tissues (biological replicates with 3-10 siliques each) were harvested at approximately 10 AM at 15, 25 and 35 days after flowering (DAF); immediately frozen in liquid nitrogen and stored at −80°C until needed. Thirty-six tissue samples were obtained at the three stages of silique development.

### Fatty acid and sugar analysis of seeds and silique walls

All samples were ground finely before freeze drying. Then, total fatty acids were extracted from approximately 30 mg dry power as detailed by Guo et al. (2017) and quantified as methyl esters through a gas chromatography-flame ionization detector (GC-FID). Heptadecanoic acid (C17:0) as the internal standard was added to the samples prior to extraction. Helium was used as the carrier gas at a constant velocity of 1.0 mL/min. The injector and FID temperatures were set at 240 and 250°C, respectively.

Soluble sugars of 15 mg dry powder were extracted and derivatized sequentially with methoxyamine hydrochloride and *N*-methyl-*N*-trimethylsilyl-trifluoroacetamide, as detailed by Zhu et al. (2021). Metabolites were quantified with an Agilent 7890 A GC/5975C MS system (Agilent Technology) on a DB-5MS capillary column (20 m × 0.18 mm × 0.18 µm) with a 5 m Duraguard column (Agilent Technology).

### RNA extraction, library preparation and high-throughput sequencing

Total RNA from 36 tissue samples was isolated using TRIzol™ Reagent (Cat. 15596026) according to the user guide, and then RNase-free DNase I (Cat. M0303S) was selected to remove contaminating genomic DNA. Construction of the cDNA library using the TruSeq Stranded mRNA LT Sample Prep Kit (Cat. RS-122-2101) and sequencing on an Illumina HiSeqTM 2500 platform were conducted following the manufacturer’s instructions at Shanghai OE Biotech Co., Ltd. (Shanghai, China). Paired-end reads with a length of 2 × 150 base pairs (bp) were generated. The database (PRJCA011946) was deposited in the National Genomics Data Center (NGDC).

### RNA-sequencing data processing, annotation and data analysis

All raw paired-end reads were filtered by Trimmomatic (Bolger et al., 2014), and then data quality was evaluated with FastQC (https://www.bioinformatics.babraham.ac.uk/-projects/fastqc). Clean reads were mapped to the rapeseed reference genome (http://plants.ensembl.org/Brassica_napus) with STAR (Dobin and Gingeras, 2016) and sorted and indexed using samtools (Li et al., 2009) to obtain the count number of the unique reads for every gene. The relative transcript abundance of target genes was normalized using the TPM (transcripts per kilobase of exon model per million mapped reads), and genes with an average TPM lower than 1 in each tissue were considered not expressed. Differentially expressed genes (DEGs) were identified using the R package EdgeR (Robinson et al., 2010) with p.adj < 0.01 and fold change ≥ 2.0.

Comparisons were designed as following: one kind of comparison was L192 versus A260 of the same tissues at the same development stage, for example, H.vs.L-S15 represented comparison between seeds of L192 (H) versus A260 (L) at 15 DAF. Another one was seeds versus silique walls of one genotype at the same development stage, for example, S.vs.W-H15 represented comparison between seeds (S) versus silique walls (W) of L192 (H) at 15DAF. The last one was comparison of different development stage of seeds or silique walls from L192 or A260, for example, 25.vs.15-HS represented comparison between seeds of 25 DAF versus 15 DAF of L192 (H).

The pathway annotation of the rapeseed genome was obtained using Mercator4 V3.0 (Schwacke et al., 2019). To obtain Gene Ontology (GO) annotation of the *B. napus* genome, blastp (e-value < 1e-40) was used to identify homologous genes against *A. thaliana* (Araport11) with the highest score. Then, GO annotations of the rapeseed genome were from Biomart (http://plants.ensembl.org/biomart/martview) and *A. thaliana* based on the BLAST results. The GO annotation database of *B. napus* was constructed by the R package clusterProfiler (Yu et al., 2012). GO enrichment analysis and gene set enrichment analysis (GSEA) were performed by clusterProfiler.

Temporal gene expression profiles were investigated using the R package ImpulseDE2 (p.adj<0.01) (Fischer et al., 2018) at 15, 25 and 35 DAF during the development of 18 seed samples or 18 silique wall samples followed by deleting genes with more than 9 missing values among 18 samples. For the gene coexpression network analysis, 15,464 DEGs were used. The log2 (TPM + 1) was used as input. All samples were classified into 24 categorical variables according to the genotype, sample time and tissue type with Boolean values (**Supplementary Table 2**). After the appropriate soft-thresholding power was computed (**Supplementary Figure 1**), network concepts were calculated *via* the blockwiseModules() function (power = 14, minModuleSize = 50, mergeCutHeight = 0.25, corType = Pearson) in the R package WGCNA (weighted gene correlation network analysis) (Langfelder and Horvath, 2008). Enrichment analysis for each module was conducted by clusterProfiler. The hub genes of each WGCNA module were calculated using the R package dhga (Das et al., 2017). Finally, the weighted gene coexpression network was visualized with Cytoscape (Shannon et al., 2003).

For gene expression patterns, all genes with expression levels < 1 TPM were set to 0, and a mean value from all replicates for each tissue separately was calculated. Means were selected to calculate the tau index (Kryuchkova-Mostacci and Robinson-Rechavi, 2017) to identify whether a gene was specifically or ubiquitously expressed. Genes with a tau index close to 1 are more specifically expressed in one sample, while genes with a tau index closer to 0 are equally expressed across all samples studied.

### Histochemical analyses

To investigate embryo development at 15, 25 and 35 days after flowering, seeds were fixed in FAA (45% Et-OH, 5% acetic acid, 5% formalin, and 45% water) and then dehydrated in 60%, 75%, 85%, and 95% Et-OH for 30 min at each concentration; in 100% xylene for 2 h, in xylene:paraffin 1:1 for 2 h and in 100% paraffin for 48 h at 58°C, and finally embedded in paraffin at 4°C for histological sections (8 µm thickness). Slices were stained with 0.1% toluidine blue and observed with a light microscope.

### Data analysis and visualization

Data were analysed and visualized using R packages such as tidyverse, ggplot2 and ComplexHeatmap (Gu, 2022). The averages and standard deviations (SD) of all results were calculated, and one-way ANOVA and multiple paired Student’s *t* tests were performed to generate p values.

## Results

### Characteristics of two *B. napus* varieties in silique development

In dry seeds, the oil contents of L192 (H) and A260 (L) were 55.82 ± 2.11% and 37.39 ± 1.53%, respectively, as detected by the NMR method. Furthermore, the contents of oleic acid (C18:1, OA), linoleic acid (C18:2, LA), linolenic acid (C18:3, ALA), erucic acid (C22:1, EA), glucosinolate (GLS) and saturated fatty acid (SFA) were significantly different between L192 and A260 based on the detection results with NIRS (**Supplementary Table 1**).

**Figure 1:**
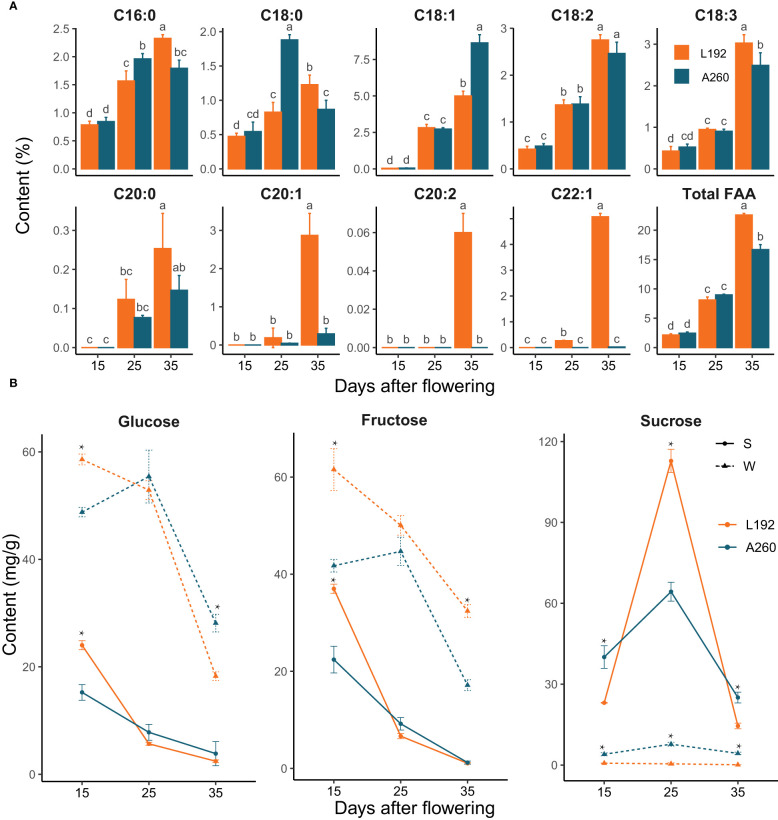
Fatty acid or sugar variability at 15, 25 and 35 DAF during silique development. **(A)** Changes in the content of different types of fatty acids in the seeds at 15, 25 and 35 DAF. The bars represent the means of three biological replicates, and error bars are standard deviations. Different letters marked on bars indicate significant differences according to *t* test analyses (p < 0.05) among groups. **(B)** The sugar content of the seed or silique wall. Significant differences between mean values were calculated at each time point using *t* test analyses and are represented by asterisks at the p < 0.05 level.

To investigate the difference in metabolic intermediates between L192 and A260 during silique development, the contents of fatty acids (FAs) and soluble sugars in seeds and silique walls were measured at 15, 25 and 35 days after flowering (DAF) (**Figure 1**). In developing seeds of both L192 and A260, the contents of FAs obviously gradually increased, especially after 25 DAF (**Figure 1A**), but not in silique walls with similar content at the different development periods (**Supplementary Figure 2**). Before 25 DAF, the contents of palmitic acid (C16:0) and stearic acid (C18:0) in A260 seeds were higher than those in L192 seeds (**Figure 1A**), but other FAs and the content of total fatty acids showed no significant difference. At 35 DAF, except OA, the contents of each FA and total FAs in L192 seeds were higher than those in A260 seeds (**Figure 1A**). Based on the above results, the difference in total FA accumulation efficiency after 25 DAF may be one reason for the significant difference in final SOC between L192 and A260.

Silique walls with photosynthetic activity play pivotal roles not only in protecting seeds from pests and pathogens by encapsulation but also in contributing assimilates and nutrients to fuel seed growth (Bennett et al., 2011). The contents of soluble sugars, including sucrose, glucose and fructose, were tested in seeds and silique walls (**Figure 1B**). In general, the contents of glucose and fructose quickly decreased, especially after 25 DAF, in the seeds and silique walls of L192 and A260, and their contents were higher in the silique walls than in the seeds at the same developmental stage. Furthermore, the contents of fructose and glucose were higher in L192 than in A260 at 15 DAF, vice versa at 35 DAF and similar at 25 DAF in silique walls (**Figure 1B**). For sucrose, the peak content occurred at 25 DAF in both L192 and A260 seeds. However, the sucrose content was maintained at a low level in the silique wall, and the sucrose content was significantly lower in L192 than in A260 during the development of silique walls. More interestingly, the seed sucrose content of L192 was approximately twofold higher than that of A260 at 25 DAF, and the sucrose content of L192 was significantly lower than that of A260 in the seeds at both 15 and 35 DAF (**Figure 1B**).

Based on the seed sections of the two varieties, the developmental stages of the embryos were similar at the same time, although the seed size of A260 (1000-grain weight was approximately 3.79 g) was larger than that of L192 (1000-grain weight was approximately 2.82 g). For instance, the embryo was in the heart stage at 15 DAF, the bent stage at 25 DAF and the mature cotyledon stage at 35 DAF (**Supplementary Figure 3**) compared with *Arabidopsis* embryo development (Le et al., 2010). These results revealed that embryos were in the cell division stage with a high content of glucose and fructose (**Figure 1B**) before 25 DAF, and then storage reserve accumulation was activated by sucrose, for which a peak (**Figure 1B**) occurred at 25 DAF.

### Global transcriptional changes in L192 were similar to those in A260 during silique development

RNA-seq technology was used to determine the difference between L192 with high oil content and A260 with low oil content at the transcriptional level during silique development. There were approximately 65,628 genes with a mean TPM greater than one in at least one tissue. Based on the sample-wide cluster analysis (**Supplementary Figure 4**), 36 samples can be grouped into two subgroups: seed and silique wall. Then, seed samples at the same development period, regardless of whether they belonged to L192 or A260, were also first clustered into one group. In the silique walls, samples from L192 were first clustered into one group, which showed that genes had similar expression profiles in L192 silique walls from different time points as well as in samples from A260 (**Supplementary Figure 4**). The results showed that the expression patterns of genes in the seed were regulated mainly by the development period and that those in the silique wall were regulated by genotype.

After pairwise comparison of three time points in A260 or L192, overrepresentation analysis of upregulated or downregulated genes showed that enriched biological processes were similar between the two genotypes during seed development (**Supplementary Figure 5**). For example, fatty acid biosynthesis, seed oil storage, seed maturation and glucosinolate catabolic processes were preferentially enriched in both genotypes based on the enrichment analysis of upregulated genes. Response to light, photosynthesis, sucrose metabolic process, lipid transport, cutin and wax biosynthetic process and plant ovule development were preferentially enriched in both genotypes based on the enrichment analysis of downregulated genes. For the developing silique wall, the transcriptional changes in L192 were also similar to those in A260 (**Supplementary Figure 6**). Apart from that, based on the tau index (more than 0.8) of each gene across 6 tissues of A260 or L192, there were approximately 8,819 genes with spatiotemporal specific expression in A260 and/or L192 during silique development, and only 335 gene expression peaks in the two genotypes appeared in the different spatiotemporal tissues (**Supplementary Table 3**). The above results showed that the gene expression patterns of L192 were similar to those of A260, and the reasons for the SOC difference between them were the difference in gene expression levels during silique development.

### The expression of genes related to lipid biosynthesis was enhanced over the period investigated

**Figure 2:**
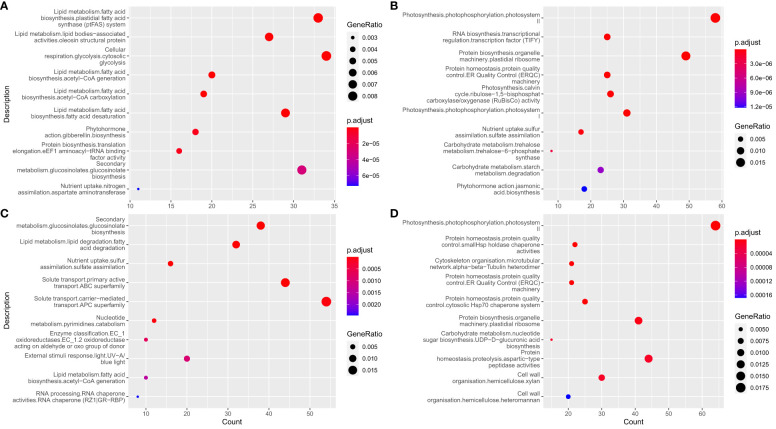
Enrichment analysis of genes with temporal expression profiles. **(A, B)** enrichment results of significantly enhanced and inhibited genes over during seed development, respectively. **(C, D)** enrichment results of significantly enhanced and inhibited genes during the development of silique walls, respectively.

The mean expression trajectory of each gene over time in L192 versus A260 at 15 DAF, 25 DAF and 35 DAF during their seed development was investigated. For the seeds, approximately 6,492 and 5,391 genes were significantly enhanced and inhibited over the period investigated, respectively. Based on Mapman (**Figure 2** and **Supplementary Table 4**) or GO (**Supplementary Figure 7** and **Supplementary Table 5**) enrichment results of enhanced genes, the expression of genes related to fatty acid biosynthesis and lipid storage, gibberellin biosynthesis, solute transport and glucosinolate biosynthesis was significantly enriched (**Figure 2A**, **Supplementary Figure 7A** and **Supplementary Table 4** **.1**). The expression of genes related to photosynthesis, starch degradation, protein homeostasis and biosynthesis was inhibited over time (**Figure 2B**, **Supplementary Figure 7B** and **Supplementary Table 4** **.2**).

In *B. napus*, approximately 3,038 genes were involved in lipid metabolism through sequence alignment (e-value ≤ 40) according to *A. thaliana* annotation (http://aralip.plantbiology.msu.edu); approximately 2,243 genes with mean of TPM from all replicates for each tissue more than one were detected in 36 samples, and 2,101 genes were detected in all 18 seed samples (**Supplementary Table 6**). The expression levels of 391 and 236 genes related to lipid metabolism were significantly enhanced and inhibited over the period, respectively (**Supplementary Table 6**).

**Figure 3:**
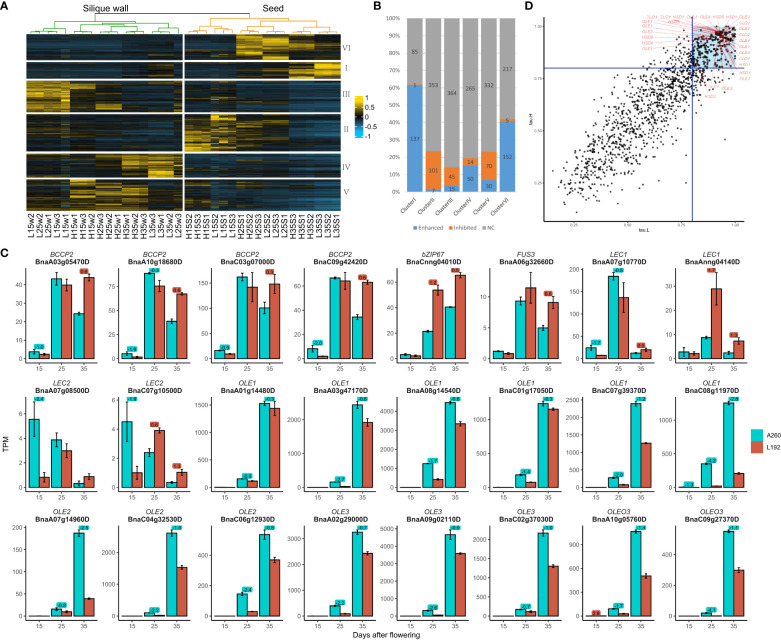
Expression of genes related to the lipid pathway in the two genotypes. **(A)** cluster analysis of genes relative to the lipid pathway (**Supplementary Table 6**). H, samples of L192 (high oil content). L, samples of A260 (low oil content). S, seed. W, silique wall. 15, 25 and 35, 15, 25 and 35 days after flowering, respectively. The number at the end of the sample name was the code number of three biological replicates. **(B)** the ratios of the enhanced or inhibited genes over the period investigated in each cluster. The numbers were counts of genes enhanced, inhibited or no change (NC) by time-course in each cluster (**Supplementary Table 6**). **(C)** Expression profiles of some important genes related to lipid biosynthesis in the seed. The bars represent means of three biological replicates (TPM value), error bars are the standard deviation, and the numbers on the bars are the logFC (FC, fold change, comparison of L192 versus A260 at 15, 25 or 35 DAF). **(D)** The tau indices of genes relative to the lipid pathway in L192 (tau.H) or A260 (tau.L). The blue lines were where the tau index was equal to 0.8. Genes with the tau index greater and equal to 0.8 are more specifically expressed in one sample (Kryuchkova-Mostacci and Robinson-Rechavi, 2017). The tau indices of the oil body proteins (**Supplementary Table 3**) were greater than 0.8 and marked with red lines. *OLE Oleosin*, *CLO Caleosin*, *HSD Hydroxysteroid dehydrogenase*. The points in the blue block represented tau indices of genes both in L192 and A260 were greater than 0.8.

Genes related to the lipid pathway were clustered into six groups, Clusters I, II, III, IV, V and VI. The expression peaks of clusters II, VI and I mainly occurred in the seeds at 15, 25 and 35 DAF, respectively, and the other three clusters mainly occurred in the silique walls (**Figure 3A**). Almost all genes relative to plastidial fatty acid synthase, such as 14 out of 16 members of *ACCase* (*α-CT*, *BC*, *BCCP1* and *BCCP2*) (**Supplementary Table 6**), belonged to cluster VI, with the expression of 152 genes (approximately 40.64%) enhanced over time (**Figure 3B** and **Supplementary Table 6**). Four *BCCP2*s were expressed at lower levels in L192 than in A260 at both 15 and 25 DAF but at higher levels in L192 at 35 DAF (**Figure 3C**). Then, Cluster I, in which 61.43% (137 out of 223) of the members were enhanced over the period investigated, covered all lipid droplet-associated genes with spatiotemporal specificity of expression, such as *OLE*, *CLO*, *HSD* and *PXG*, expressed in the seed at 35 DAF according to the tau index (**Figure 3D** and **Supplementary Table 6**). However, all lipid droplet-associated genes were expressed at lower levels in L192 with a high oil content than in A260 (**Supplementary Table 6**). For example, all 14 *OLEs* were expressed at significantly lower levels in L192 than in A260 at both 25 DAF and 35 DAF (**Figure 3C**). The results revealed that Cluster I, mainly for the formation of oil bodies, and VI, for the biosynthesis of *de novo* fatty acids, were important for seed lipid storage during seed maturation.

### Differences in photosynthetic capacity between the two genotypes

For the silique walls, which contribute photosynthetically assimilated carbohydrates and nutrients to fuel seed development (Bennett et al., 2011; Zhu et al., 2018; Li et al., 2019), the expression of approximately 4,948 and 5,676 genes was significantly enhanced and inhibited over the period investigated, respectively. The enhanced genes were mainly involved in pathways such as glucosinolate biosynthesis, fatty acid degradation and solute transport (**Figure 2C**, **Supplementary Figure 7C** and **Supplementary Table 7.1**, **8.1**), while genes related to photosynthesis, protein homeostasis, cytoskeleton and cell wall organization were inhibited over the period investigated (**Figure 2D**, **Supplementary Figure 7D** and **Supplementary Table 7.2**, **8.2**).

**Figure 4:**
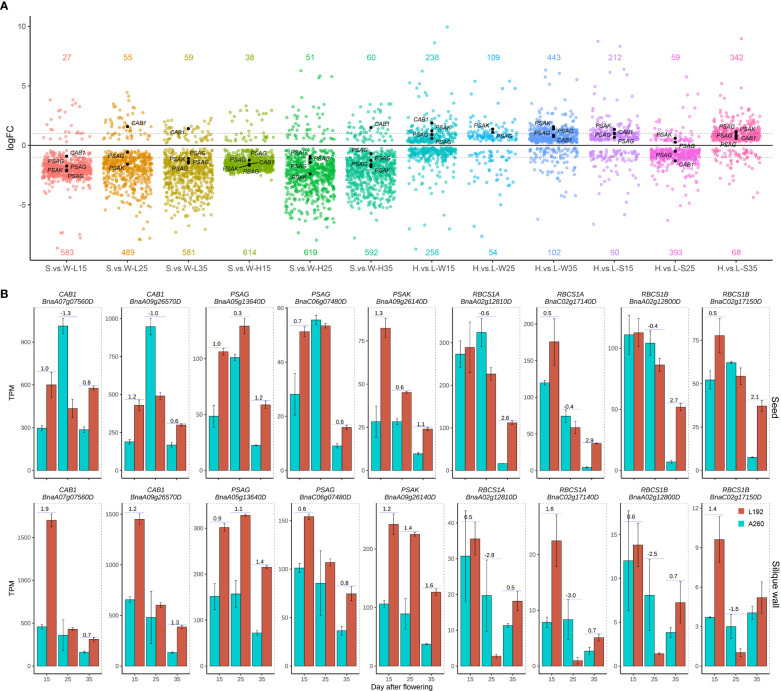
Expression difference of photosynthesis genes between the two genotypes. **(A)** Quantity variance of DEGs between seeds and silique walls in one genotype or DEGs of L192 versus A260 in seeds or silique walls. The number represents the number of upregulated (above the x-axis) or downregulated (below the x-axis) genes. One point represents one DEG. H, samples of L192 (high oil content). L, samples of A260 (low oil content). S, seed. W, silique wall. 15, 25 and 35, 15, 25 and 35 days after flowering, respectively. H.vs.L-S, comparsion of L192 versus A260 in the seeds. H.vs.L-W, comparsion of L192 versus A260 in the silique walls. S.vs.W-L, comparsion of seeds versus silique walls of A260. S.vs.W-H, comparsion of seeds versus silique walls of L192. **(B)** Expression profiles of some photosynthesis related genes. The bars represent the means of three biological replicates (TPM value), error bars are the standard deviation, and the numbers on the bars are the logFC (FC, fold change, comparison of L192 versus A260). *CAB*, Chlorophyll A/B binding protein; *PSAK*, Photosystem I subunit K; *PSAG*, Photosystem I subunit G; *RBCS1A*, Rubisco small subunit 1A; *RBCS1B*, Rubisco small subunit 1B.

According to MapMan annotations, approximately 864 genes were related to photosynthesis (**Supplementary Table 9**), and approximately 743 genes were detected with a maximum TPM greater than 1 according to the RNA-seq results from 36 samples. High expression of most genes occurred not in seeds but in silique walls (**Figure 4A**; **Supplementary Figure 8**). According to the gene expression difference between the seed and silique wall (**Supplementary Table 9**), approximately 243 genes were more highly expressed in the silique wall than in the seed at all three stages of silique development at the same stage, and 375 genes were more highly expressed in the silique wall at the different stages in the two genotypes. These results suggested that silique walls had more photosynthetic activities than seeds at the same stages. However, approximately 77 genes had significantly higher expression in the seeds than in the silique walls at all or some stages in L192 and/or A260 (**Supplementary Table 9**). For example, four small subunits of RubisCO, *RBCS1A* (BnaA02g12810D and BnaC02g17140D), *RBCS1B* (BnaC02g17150D) and *RBCS3B* (BnaA02g12800D) (**Figure 4B**), were more highly expressed in the seed than in silique walls at all three stages of L192 and A260. RubisCO, which is the rate-limiting step that decides the efficiency of the fixation process, has important roles in improving the carbon efficiency of developing green seeds, producing approximately 39% phosphoglycerate in rapeseed embryos (Schwender et al., 2004a). In addition, the expression of four small subunits of RubisCO in seeds of L192 was more than 2-3 times higher than that of A260 at 35 DAF (**Figure 4B**; **Supplementary Table 9**), which suggested that L192 had higher carbon efficiency than A260.

More interestingly, the expression levels of genes related to photosynthesis were higher in L192 with high SOC than in A260 with low SOC during the development of silique walls at 25 DAF (109/54, the number of genes with higher expression in L192 and A260, respectively), 35 DAF (443/102) and in seeds at 15 DAF (212/90) and 35 DAF (342/68) (**Figure 4A**). For example, light-harvesting complex II *CAB1* (BnaA09g26570D and BnaA07g07560D) and photosystem I reaction centre subunit *PsaG* (BnaC06g07480D and BnaA05g13640D) and *PsaK* (BnaA09g26140D) had higher expression levels in the seeds of L192 than in those of A260 (**Figures 4A, B**), suggesting that L192 seeds can catch more light and produce more reductives for biosynthesis than A260 seeds. And candidate genes related to photosynthesis were screened, and might improve the seed oil content of rapeseed according to expression changes between L192 and A260 especially at 35 DAF in the seed (**Supplementary Tables 10**).

**Figure 5:**
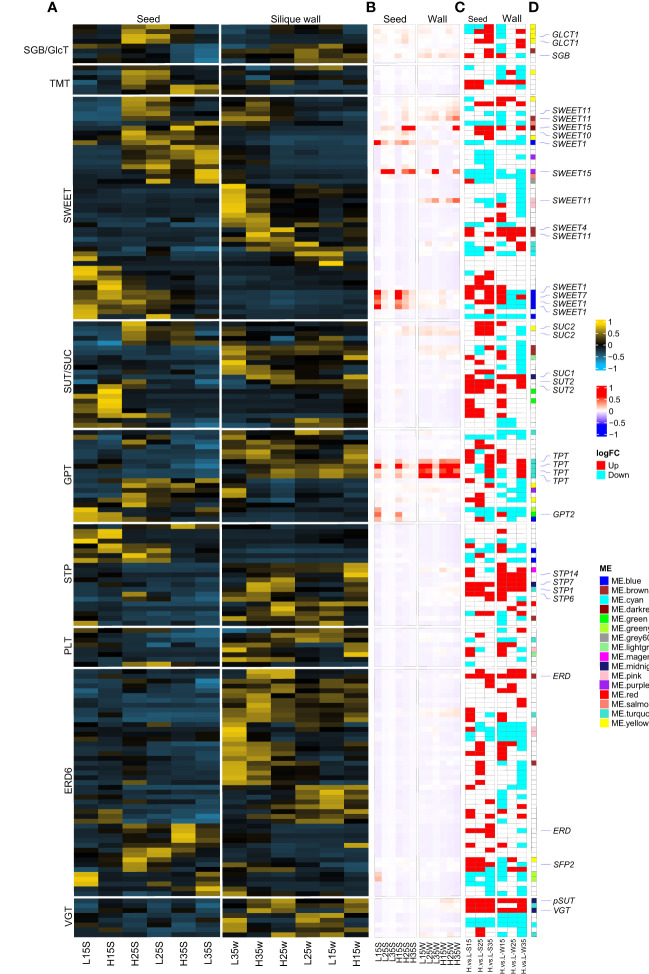
Differences of sugar transporters between L192 and A260. **(A, B)** heatmap of sugar transporters at the sample-wide level or gene-wide level according to the TPM mean of three biological replicates, respectively. **(C)** Difference in the expression of L192 versus A260. H, samples of L192 (high oil content). L, samples of A260 (low oil content). S, seed. W, silique wall. 15, 25 and 35, 15, 25 and 35 days after flowering, respectively. H.vs.L-S, comparsion of L192 versus A260 in the seeds. H.vs.L-W, comparsion of L192 versus A260 in the silique walls. **(D)** Sugar transporters belonging to some modules according to WGCNA. Sugar transporters marked in the white block did not belong to any modules. VGT, vacuolar glucose transporter. ERD6, early response to dehydration 6, encodes a putative sucrose transporter whose gene expression is induced by dehydration and cold. PLT, polyol/monosaccharide transporter. STP, sugar transporter. GPT, glucose-6-phosphate/phosphate translocator. SUT/SUC, sucrose transporter/sucrose-proton symporter. SWEET, Sugar Will Eventually be Exported Transporter. TMT, tonoplast monosaccharide transporter. SGB/GlcT, suppressor of G protein beta/plastidic glycose translocator. TPT, triose-phosphate/phosphate translocator. pSUT, plastidic sugar transporter.

The delivery of photoassimilates from source tissues into the sink seed or translocation of sugar among subcellular organelles by means of sugar transporters was investigated. Among soluble sugar transporters, GPTs (G6P/phosphate translocator), SUT/SUCs and SWEETs might play more important roles in seed development because their expression was higher at the gene-wide levels (**Figure 5A**) or sample-wide expression levels in the seeds (**Figure 5B**). For example, at the gene-wide level in developing seeds, homologous genes SWEET1 (BnaA06g15180D, BnaA08g21340D, BnaC05g16660D and BnaCnng57120D), SWEET10 (BnaA03g13530D), and SWEET15 (BnaC02g04530D, BnaA02g01450D and BnaC03g71480D) were highly expressed in both L192 and A260. Then, homologous genes of TPT/APE2 (BnaA02g24310D, BnaA06g36180D, BnaC02g47130D and BnaC07g18860D) and SWEET11 (BnaC08g20440D, BnaC03g52910D and BnaA06g16330D) were highly expressed in the silique walls (**Figure 5A**). However, compared with A260, approximately 60% of sugar transporters were expressed more highly in L192 seeds, especially at 35 DAF (**Figure 5C**). This revealed that L192 had a stronger ability to transport soluble sugars.

### Gene set enrichment analysis showed the difference in the biosynthesis of storage reserves between L192 and A260 in immature seeds

**Figure 6:**
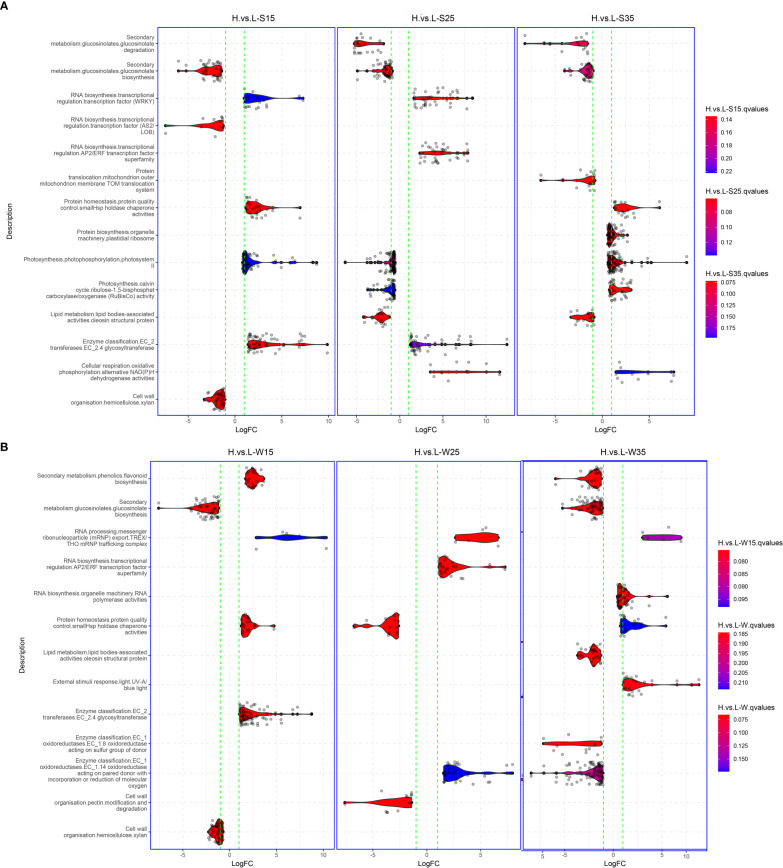
Gene set enrichment analysis between L192 and A260. **(A)** Different pathways at 15, 25 and 35 DAF in the developing seed. **(B)** Different pathways at 15, 25 and 35 DAF in the developing silique walls. Gene sets were obtained using Mercator4 (Schwacke et al., 2019).

The differences between L192 and A260 were compared at the transcriptional level in seed development, and approximately 20,005, 26,512 and 24,968 genes with differential expression were screened in seeds at 15, 25 and 35 DAF, respectively (**Supplementary Tables 11**, **12**, **13**). To investigate which biological pathways are the possible causes of the differences in seed storage reserve synthesis between L192 and A260 during the seed development process, gene set enrichment analysis (GSEA) was performed. According to the GSEA results (**Figure 6**), there were approximately 60 and 68 enriched pathways in the seeds (**Figure 6A**, **Supplementary Table 14**) and silique walls (**Figure 6B**, **Supplementary Table 15**) during their development at the three time points, respectively.

Compared with the mature seed of A260, L192 had a higher oil content. However, three gene sets relative to lipid metabolism, such as diacylglycerol biosynthesis, oleosin structural protein and caleosin lipid body surface protein, were enriched only at 25 DAF, and the first was active and while the latter two were inhibited in L192 seeds (**Figure 6A**, **Supplementary Table 14**). Interestingly, in L192 seeds, the pathways related to photosynthesis, such as photosystems I and II and Rubisco activity, were enhanced at 15 and/or 35 DAF, while photosystem II and Rubisco activity were inhibited at 25 DAF (**Figure 6A**; **Supplementary Table 14**). In addition, in the process of silique wall development, gene sets relative to photosynthesis were also enriched, such as chlororespiration, photosystem I, II and Rubisco activity, which were more enhanced at 35 DAF (**Supplementary Table 15**) in L192. Based on the above results, the expression of genes related to photosynthesis was the most variable both in the seed and silique wall between the two genotypes, and this may be the main cause of the difference in lipid accumulation between L192 and A260.

**Figure 7:**
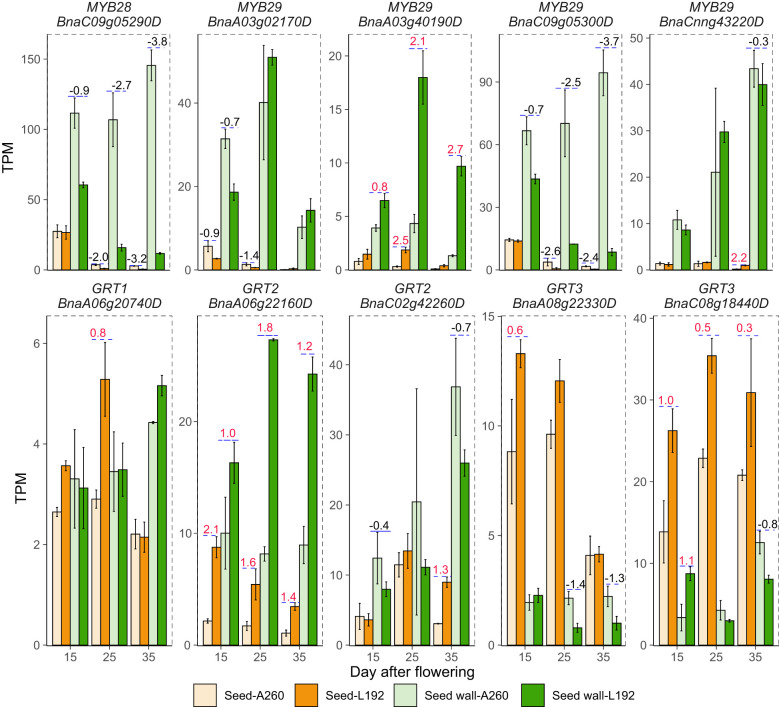
Expression of the main genes relative to glucosinolate between L192 and A260. The bars represent the means of three biological replicates (TPM value), error bars are the standard deviation, and the numbers on the bars are the logFC (FC, fold change, comparison of L192 versus A260). 15, 25 and 35, 15, 25 and 35 days after flowering, respectively.

GLS content is an important seed quality trait for *B. napus* breeding. The concentration of L192 (48.01 ± 1.34 µmol/g) was lower than that of A260 (139 ± 11.6 µmol/g) in the mature seeds (**Supplementary Table 1**). According to the RNA-seq results, during seed development, the glucosinolate biosynthesis pathways of L192 seeds were inhibited at the three time points compared with those of A260 seeds, and the GLS biosynthesis pathway in silique walls of L192 was also inhibited at 15 and 35 DAF compared with the pathway in silique walls of A260 (**Figure 6B**; **Supplementary Table 15**). Compared with A260, the expression of most genes related to glucosinolate biosynthesis was downregulated in seeds or silique walls of L192 at the same stages (**Supplementary Table 15**). MYB28 and MYB29 are the mast regulatory factors of GLS content and positively control the biosynthesis of aliphatic GLS in *Arabidopsis* (Gigolashvili et al., 2007; Gigolashvili et al., 2008). Based on RNA-seq, *BnMYB28* (BnaC09g05290D, only one copy) and four out of seven *MYB29*s (BnaA03g02170D, BnaA03g40190D, BnaC09g05300D and BnaCnng43220D) were detected and highly expressed in seed siliques, and the expression of *BnMYB28* was significantly lower in five tissues from L192 than in A260 at different stages (**Figure 7**). Meanwhile, BnaC09g05300D was highly expressed in samples from L192 (**Figure 7**). Apart from that, GRT1, GRT2 and GRT3 play important roles in long-distance transport of aliphatic GLS or import of indole GLS into storage cells (Nour-Eldin et al., 2012; Jørgensen et al., 2017; Nour-Eldin et al., 2017). In *B. napus*, there were approximately 6, 8 and 6 members of GRT1, GRT2 and GRT3, respectively (**Supplementary Table 16**). Only 5 *GRT*s, such as 1 *GRT1*, 2 *GRT2*s and 2 *GRT3*s, were more highly expressed in some samples from L192 than A260 (**Figure 7**). The expression levels of 2 *GRT3*s, BnaA08g22330D and BnaC08g18440D, were higher in seeds than in silique walls between L192 and A260, and BnaC08g18440D (*GRT3*) was more highly expressed in the seeds of L192 than in those of A260 at the three stages (**Figure 7**). Additionally, the expression of BnaA06g22160D (*GRT2*) was higher in the seeds of L192 than in those of A260 and was verified to reduce the seed GLS content in knockout mutants (He et al., 2022; Tan et al., 2022). In addition, the two *GRT3* genes highly expressed in seeds may be selected as candidates for improving GLS breeding in *B. napus* based on our results.

### Multiple modules synergistically regulated seed development

**Figure 8:**
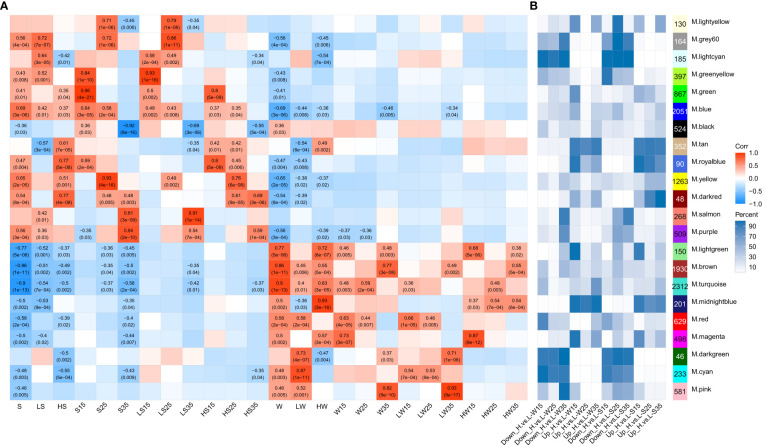
Correlations between module eigengenes and spatiotemporal traits of samples. **(A)** the module–trait associations. The numbers within the heatmap represent correlation coefficients and *p* values (in parentheses; red, positively correlated, blue, negatively correlated) for the module–trait associations. **(B)** distributions of each module in comparisons. This number is the number of genes from each module as a percentage of the total number of genes per module. Down_H.vs.L and Up_ H.vs.L, down- and up-regulated genes in L192 versus A260, respectively. S, seed. W, silique wall. 15, 25 and 35, 15, 25 and 35 days after flowering, respectively.

Based on the edgeR results of pairwise comparisons among 24 groups generated by the same tissues with different stages and the same stages with the different tissues within or across the cultivars, approximately 15,464 genes with a max TPM greater than 10 among the 36 samples were selected to construct coexpression networks during the development of siliques through WGCNA (weighted gene coexpression network analysis). Finally, approximately 13,428 genes were grouped into 22 distinct modules (**Figure 8A**; **Supplementary Table 16**). The module eigengene (first principal component for each module) can be considered a representative of the gene expression profiles in a module (Langfelder and Horvath, 2008), and the positive correlation indicated that the module had higher expression in the given tissue than in all other tissues. Among the 22 modules, 12 modules were significantly positively correlated with the different stages of the seed, and 9 modules were significantly positively correlated with the different stages of the silique wall (**Figure 8A**). The first two largest modules, ME.turquoise and ME.blue, were mostly correlated with the silique wall (r = 0.9, p value = 1e-13) and seed (r = 0.69, p value = 3e-6), respectively. For the seeds, ME.blue had a significantly positive correlation with all of the seed samples except for seeds at the 35 DAF. ME.green, ME.yellow and ME.purple were positively correlated with the seeds at 15 DAF (r = 0.96, p value = 4e-21), 25 DAF (r = 0.93, p value = 4e-16) and 35 DAF (r = 0.84, p value = 2e-10), respectively. The three modules showed a higher correlation with L192 seeds than with A260 seeds. In addition, ME.salmon (r = 0.91, p value = 1e-14) exhibited a significantly positive correlation with A260 seeds at 35 DAF. For the silique wall, in addition to ME.turquoise, ME.brown, the third largest module, was related to silique walls (r = 0.86, p value = 1e-11) and showed a significantly positive correlation with the siliques of both L192 and A260 at 35 DAF. However, ME.pink showed a significantly positive correlation only with the A260 siliques at 35 DAF. Our findings suggested that those modules not only played important roles in the development of the seed and the silique wall but also suggested the difference in the development of the seed and the silique wall between L192 and A260.

The expression profiles of members of some modules were also related to genotype according to the distribution of most modules among the six comparison groups (L192 versus A260) (**Figure 8B**, **Supplementary Table 17**). For instance, the members of four modules, ME.darkred, ME.tan, ME.midnightblue and ME.royalblue, were preferentially expressed in L192. However, the members of five modules, ME.darkgreen, ME.lightcyan, ME.cyan, ME.grey60 and ME.greenyellow, were strongly expressed in A260. In addition, members of some modules also exhibited stage-specific expression in the seed or silique wall. More than half of the members of ME.salmon, ME.purple and ME.pink exhibited high transcript abundance in silique walls at 35 DAF and in A260 seeds 25 and 35 DAF, respectively. Approximately 50% of the members of ME.green and ME.yellow had higher levels of transcription in L192 seeds than in A260 seeds at 15 and 25 DAF, respectively. Overall, these findings confirmed that these modules played important roles in the difference in seed or silique wall development.

**Figure 9:**
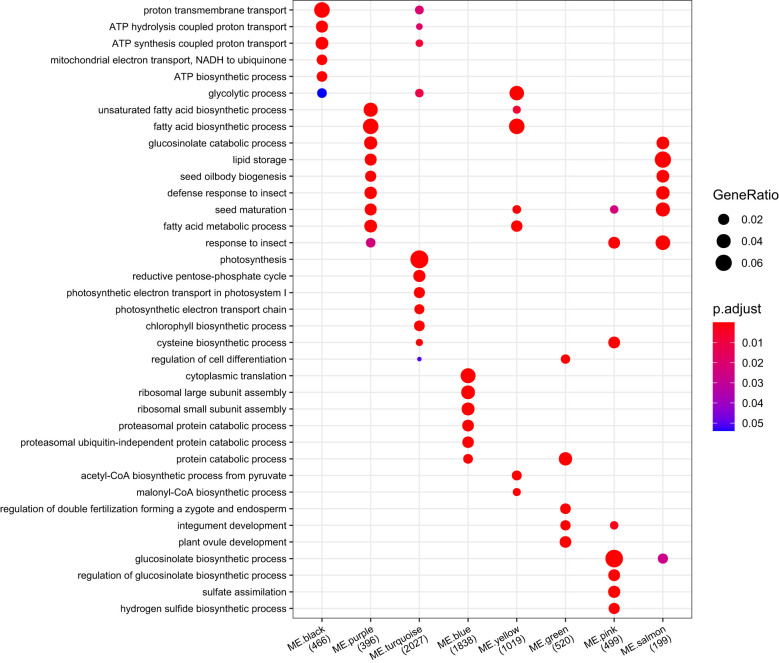
Comparing GO functional profiles of 22 module eigengenes. The top 3 GO enriched biological process of each module were displayed, and other results were listed in **Supplementary Table 18**. The numbers under each module were counts of genes annotated by GO.

To enrich the main biological process/pathway of modules, an overrepresentation test was implemented in clusterProfiler based on the GO and MapMan annotation (**Supplementary Table 18**). The biological process/pathway significantly enriched (p < 0.05) in each module can represent the biological function mainly involved by each module (**Figure 9**, **Supplementary Table 18** **.1**). Photosynthesis was the most significantly enriched pathway in ME.turquoise (q value = 9.23E-115), and the biological functions of ME.turquoise were mainly related to light harvesting, photosynthetic electron transport, photophosphorylation of photosystems I and II, and chlorophyll biosynthetic processes (**Figure 9**, **Supplementary Table 18.1**). Glucosinolate biosynthesis, amino acid biosynthetic process and protein biosynthesis were preferentially enriched in ME.pink, ME.brown and ME.blue, respectively (**Figure 9**). ME.green may play important roles in the development of embryos (**Figure 9**), and the enriched GO biological processes were regulation of double fertilization, regulation of cell differentiation and plant ovule development. Acetyl-CoA generation, acetyl-CoA carboxylation and plastidial fatty acid synthase during the processes of fatty acid biosynthesis and the glycolytic process were significantly enriched in ME.yellow (**Figure 9**). For ME.purple, its biological functions were involved in fatty acid desaturation, seed oil body biogenesis and seed maturation (**Figure 9**). The biological function of ME.salmon was similar to that of ME.purple, except for fatty acid biosynthesis (**Figure 9**). Overall, ME.turquoise, ME.brown, ME.green, ME.yellow, ME.purple, ME.salmon and ME.pink were the main regulation modules of silique development and synergistically affected the development of rapeseed in terms of photosynthesis, embryo development and the accumulation of storage material.

**Figure 10:**
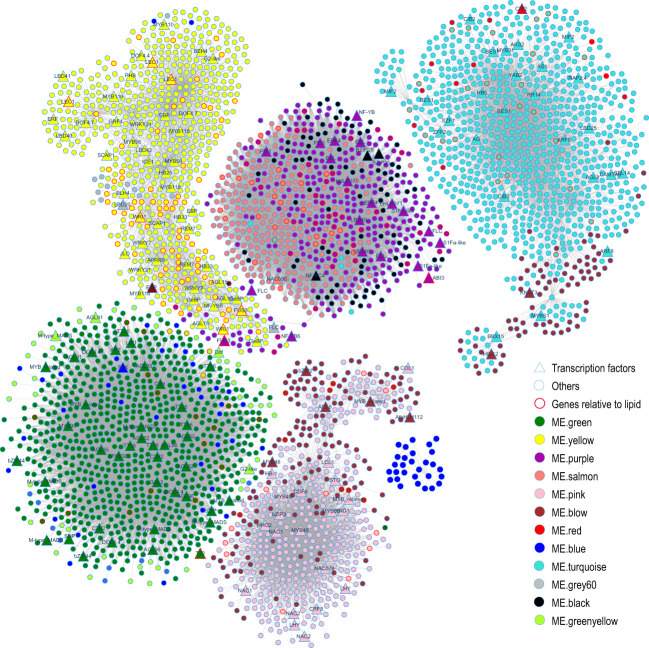
Coexpression network during silique development. Gene names of transcription factors were shown in the figure. And gene names are names of their *A. thaliana* homologs (**Supplementary Table 16**).

To identify hub genes of each module from the WGCNA results, the R package dhga v0.1 (Das et al., 2017) was used to screen the top 30 hub genes of each module (**Supplementary Table 16**). Then, the visualizing coexpression networks of the hub genes of ME.turquoise, ME.brown, ME.green, ME.yellow, ME.purple, ME.salmon and ME.pink and their associated genes were constructed by Cytoscape v8.3 (Shannon et al., 2003) according to the coexpression network WGCNA (**Figure 10**). The functions of genes associated with the hubs of modules confirmed the biological function of each module. For instance, ME.green contained 9 genes belonging to the type I MADS-box family, such as *AGL35* (2/2), *AGL62* (3/3), *AGL91* (1/1) and *AGL96* (3/3) (the number of genes in one ME/total number of genes in 22 MEs) (**Figure 10**, **Supplementary Table 16**), and they play a dual role in regulating early embryo or endosperm development in *A. thaliana* (Zhang et al., 2018). ME.yellow contained some known important transcription factors of fatty acid biosynthesis, such as homologous genes of *WRI1*(2/3), *LEC1* (3/3), *L1 L* (1/3), *AGL15* (3/3), *AGL18* (1/2) and *FUS3* (1/2) (**Figure 10**, **Supplementary Table 16**), which positively regulate biosynthesis of fatty acids during seed development in *A. thaliana* (Santos-Mendoza et al., 2008; Baud and Lepiniec, 2010; Li-Beisson et al., 2013), and *BC* (4/4), *BCCP1* (1/3) and *BCCP2* (4/4) (**Figure 10**, **Supplementary Table 16**), which are the key subunits of ACCase, a key rate-limiting enzyme for fatty acid synthesis. Therefore, ME.yellow plays important roles in the biosynthesis of fatty acids in the seed. *FAD3* (4/5), *HSD1* (1/4), *HSD6* (1/1), *CLO1* (2/3), *OLE1* (4/6), *OLE2* (1/3), *OLE4* (3/3), *ABI3* (2/2), *FUS3* (1/2), *bZIP67* (2/2) and *DGAT1* (1/1) were members of ME.purple (**Supplementary Table 16**). ME.salmon covered *CLO1*(1/3), *OLE1*(2/6), *OLE2* (2/3), *OLE3* (2/2), *HSD1* (3/4), and *HSD5* (2/2) (**Supplementary Table 16**); the first four are related to seed oil body biogenesis, and the last two are related to fatty acid desaturation. In addition to oleosins, ME.purple and ME.salmon also covered all of the seed storage proteins (**Supplementary Table 16**). Additionally, some important genes related to glucosinolate biosynthesis, such as *MYB28*, *MYB29*, *MAM1*, *AOP1*, *AOP3* and *GRT2*, belonged to ME.pink or ME.brown (**Supplementary Table 16**). These results revealed that ME.pink, ME.brown, ME.purple and ME.salmon played more important roles in the accumulation of storage reserves in the seed.

## Discussion

Enhancing photosynthesis to some extent is widely accepted as critical to advancing crop yield, and it provides the basic assimilated carbons for storage reserves, such as oil, protein and starch, in storage organs. Conversions of photosynthate to seed storage reserves are crucial to plant fitness and agricultural production in seed crops (Goffman et al., 2005). For rapeseed, light reaching embryos not only provides enough energy to produce the reducing agents and ATP required for fatty acid biosynthesis, but also induces expressions of genes required for storage product biosynthesis in embryos (Tan et al., 2020). Additionally, the starch disappears during oil accumulation for starch synthesis and degradation occurring simultaneously during embryo development (Da Silva et al., 1997; Andriotis et al., 2010). In *B. napus* embryos, the dominant metabolic flux is the conversion of sugars into TAG (Schwender et al., 2004b), resulting in more than 60% of the carbon being stored as oil (approximately 45% of dry weight) (Schwender et al., 2004a). Based on transcriptome of developing seeds and silique wall tissues of two contrasting inbred lines with ~13% difference in seed oil content, genes related to photosynthesis, carbohydrates, transporters, and triacylglycerol and fatty acid synthesis tended to be upregulated in the high-oil-content line (Shahid et al., 2019). Furthermore, SOC is one of the most important agronomic traits and is related to the photosynthesis of seeds and silique walls and is independent of leaf photosynthesis and phloem transport during oil accumulation but requires metabolic influx mainly from the silique wall (Hua et al., 2012; Tan et al., 2015). Therefore, investigation of the relationship between photosynthesis in silique walls and lipid accumulation in seeds is of great importance to improve SOC during the silique development stage of *B. napus*.

Silique walls are the main photosynthetic organs and provide photoassimilates as a source of carbon and energy to fuel seed growth and development, although seeds with chlorophyll have the capacity for photosynthesis in *B. napus* after flowering (King et al., 1997; King et al., 1998; Hua et al., 2012; Ludewig and Flügge, 2013; Tan et al., 2015). Glucose and fructose are precursors for the biosynthesis of sucrose. In silique walls, photosynthate is mainly partitioned into sucrose (King et al., 1997), which represents the major transport form of photosynthetically assimilated carbohydrates in plants (Ward et al., 1998). Following long-distance transport, sucrose is released into seeds, where it enters cells as the main source of carbon and energy for storage reserves. Therefore, the biosynthesis and transport of sucrose in silique walls may play an important role in the accumulation of seed oil in rapeseed. Previous research showed that the expression of most genes related to photosynthesis at 15 DAF is significantly higher than that at 25 DAF in siliques (Huang et al., 2017). In this study, during the silique development of oilseed rape, the hexose content in silique walls was higher than that in seeds, and the sucrose content was lower than that in seeds. According to our results (**Figure 2D**), the expression of genes related to photosynthesis was inhibited over the period investigated. However, compared with silique walls of A260, photosynthesis pathways were more enriched and activated in those of L192 at 15 and 35 DAF (**Figure 6A** and **Figure 6B**), suggesting that L192 silique walls had stronger capacities for photosynthesis, including sucrose biosynthesis, than A260 at 15 and 35 DAF. However, the sucrose concentrations of A260 were approximately 5.8 to 39.09 times greater than those of L192 in silique walls during silique development at 15, 25 and 35 DAF (**Figure 1B**). On the other hand, the expression of sugar transporter genes was lower in A260 (**Figure 5C**) than in L129 over the period investigated, suggesting that more sucrose was not transported but remained in the silique walls of A260. Consistent with the gene expression mode, the sucrose content was significantly lower in L192 than in A260 during the development of silique walls (**Figure 1B**). These results suggested that there should be higher photosynthetic abilities in silique walls and sucrose transport of the high-oil variety L192 than that of the low-oil variety A260 to the seeds under the same conditions.

In plants, sucrose is the major form of carbohydrates transported from source tissues to sink tissues through sugar transporters. Sucrose transport is essential for the distribution of carbohydrates in plants (Truernit, 2001). Sugar transporters, such as the SUT/SUC (Sucrose Transporter) and SWEET (Sugars Will Eventually be Exported Transporter) families, play an essential role in seed development and storage reserves. Seed filling in *A. thaliana* requires *SWEET11*, *12* and *15*, which exhibit specific spatiotemporal expression patterns in developing seeds, and only the seeds of *sweet11;12;15* triple mutants showed a wrinkled phenotype (Chen et al., 2015). In maize (*Zea mays*), rice (*Oryza sativa*), soybean (*Glycine max*) or tomato (*Solanum lycopersium*), mutants of *Arabidopsis* homologous SWEET4, 11 or 15 are defective in seed filling or seed size (Sosso et al., 2015; Ma et al., 2017; Yang et al., 2018; Wang et al., 2019; Miao et al., 2020; Ko et al., 2021). During soybean domestication and improvement, *GmSWEET39* (homologue of *AtSWEET15*) is selected to increase seed oil content (Miao et al., 2020). SUC5 supplies *Arabidopsis* embryos with biotin and affects triacylglycerol accumulation (Pommerrenig et al., 2013). The *OsSUT2* mutation significantly decreased sugar export ability from leaves to seeds, with a significant reduction in tiller number, plant height, and 1,000-grain weight (Eom et al., 2011). Overexpression of the *SUT1* transporter in peas (*Pisum sativum*) can enhance sucrose phloem loading and carbon movement from source to sink, improve photosynthesis rates, and ultimately result in improved seed yields and nutritional quality in legumes (Lu et al., 2020). In the *B. napus* genome, there are approximately 22 and 68 members of the SUT/SUC and SWEET gene families, respectively (Jian et al., 2016). The expression of detected *SUT/SUC*s is lower in developing seeds, but *BnSWEET10* and *15* are abundantly expressed in developing seeds based on qRT−PCR results (Jian et al., 2016). Based on our results (**Figure 5A** and **Supplementary Table 19**), the expression peaks of *SUC2* and *BnSWEET1*, *10* and *15* occurred in the developing seeds, while those of *BnSWEET4* and *BnSWEET11* occurred in the silique walls. Generally, the transcription levels of most *BnSWEET*s were higher than those of *BnSUC*/*SUT*s in the developing seeds, and approximately 60% of sugar transporter genes were more highly expressed in the seeds of L192 than in the seeds of A260.

Through sugar transporters, sugar in the source tissues provides the carbon skeleton of biosynthesis of storage reserves for sink tissues such as seeds. Apart from that, sugar can affect the accumulation of embryo reserves. In *B. napus* embryos, the dominant metabolic flux is the conversion of sugars into triacylglycerols (Schwender et al., 2004b), resulting in more than 60% of the carbon being stored as oil (Schwender et al., 2004a). Canola embryos cultured on sucrose develop photoheterotrophic plastids that function in storage, whereas embryos germinate and become photoautotrophic without sucrose (Johnson et al., 1997). Therefore, sucrose content may be an important factor that affects the storage level of lipids. At 25 DAF, the sucrose content in the seed of L192 was approximately twice as high as that of A260 (**Figure 1B**), and embryos of both L192 and A260 were at the early-middle stage of cotyledon development (**Supplementary Figure 3**). The maximum starch content of embryos occurs at approximately 26 days after anthesis and then gradually decreases until it disappears during the mature stage of seed during oil accumulation (Da Silva et al., 1997). The hexose content rapidly fell as embryos progressed from the early- to late-cotyledon developmental stages (King et al., 1997), which was confirmed by our results (**Figure 1B**). During early cotyledon developmental stages, the total content of fatty acids began to increase rapidly after approximately 20 DAF (King et al., 1998) and was lower in L192 than A260 at 25 DAF, whereas it was significantly higher in L192 than A260 at 35 DAF (**Figure 1A**).

Gene coexpression analysis is a powerful data mining method for reconstructing the molecular regulation network at the whole genome level. More hub genes were found, and their regulatory network of some biological processes was restructured. For example, in developing seeds, six coexpression modules associated with soybean seed oil content were identified, and three genes (*GmABI3b*, *GmNFYA* and *GmFAD2-1B*) among 124 candidate genes potentially affecting soybean seed oil content were shown to control oil and fatty acid content through the integration of differential expression and coexpression analysis (Yang et al., 2019). Based on comparative transcriptomic analysis between ABA synthesis-deficient mutants (*vp5*) and normal maize (*Vp5*), one module of eight DEG coexpression modules was negatively correlated with the ABA content in *vp5* embryos, and its hub genes encoded thiamine, NRT1/PTR FAMILY proteins, calmodulin, metallothionein, and so on. Transcriptome and WGCNA analyses revealed significant ABA-related changes in metabolic pathways and DEGs between *vp5* and *Vp5* during maize seed maturation (Niu et al., 2022). In our study, comparative transcriptomic analysis identified 15,464 DEGs between both genotypes, 13,428 DEGs were clustered into 22 modules (**Supplementary Table 19**), and each module had its own unique biological function based on the enrichment analysis. For instance, ME.turquoise was related to photosynthesis, ME.black was related to energy metabolism, ME.blue was related to protein biosynthesis, ME.green was related to embryo development, ME.yellow was related to fatty acid biosynthesis, ME.purple was related to fatty acid metabolism, ME.salmon was related to lipid storage, and ME.pink was related to glucosinolate biosynthesis (**Figure 9**). Moreover, approximately 57.46% and 88.81% of members of ME.salmon, 61.89% and 55.6% of members of ME.purple, and 58.52% and 55.59% of members of ME.pink were expressed more highly in seeds of A260 than in seeds of L192 at 25 and 35 DAF, respectively (**Supplementary Table 17**). A total of 72.81% of members of ME.turquoise exhibited higher expression levels in the silique walls of L192 than in those of A260 at 35 DAF, and the numbers of upregulated genes in ME.yellow increased more through seed development in L192 than in A260 (**Supplementary Table 17**). These results suggested the main modules synergistically might regulate the metabolic flux for the biosynthesis of oil finally stored in the seed.

In conclusion, during the silique development of oilseed rape, the content of hexose in silique walls was higher than that in seeds, and the content of sucrose was lower than that in seeds, which could result from the transportation of sucrose from silique walls to seeds. Before 35 DAF, expressions of genes related to fatty acid biosynthesis were enhanced during the seed development, and expressions of genes related to photosynthesis was gradually inhibited during silique development. The expression abundances of genes related to photosynthesis but not lipids were higher in the seeds and silique walls of L192 with a high oil content than in those of A260 with a low oil content at both 15 and 35 DAF. Furthermore, the expression of sugar transporter genes was higher in L192 than in A260. High capabilities of photosynthesis and sugar transport can provide more photosynthate for seed development and push the conversion of sugars into TAG in the seeds, which will be worth to carrying out more detailed physiological experiment of photosynthetic activity and metabolite experiment in the future. Meanwhile, there might be approximately 7 main modules with different functions, such as ME.turquoise related to photosynthesis, ME.black related to energy metabolism, ME.blue related to protein biosynthesis, ME.green related to embryo development, ME.yellow related to lipid biosynthesis, ME.purple related to fatty acid metabolism and ME.salmon related to lipid storage, synergistically regulating the metabolic flux to enhance the biosynthesis of fatty acids and TAG storage in the seed.

## Data availability statement

The original contributions presented in the study are publicly available. This data can be found here: NGDC, PRJCA011946.

## Author contributions

XG, NY, LL and CF designed the research and conducted the experiments. CF and XG analysed the data and wrote the manuscript. XY, YC, and YZ reviewed the manuscript. ZH supervised the work and approved the manuscript for publication. All authors contributed to the article and approved the submitted version.
