# Supplementary material for: Transcriptomic comparison of seeds and silique walls from two rapeseed genotypes with contrasting seed oil content
Source: Front Plant Sci. 2023 Jan 13;13:1082466. doi: 10.3389/fpls.2022.1082466 (PMC9880416; doi:10.3389/fpls.2022.1082466)
Supplement: Supplementary file 1 [file DataSheet_1.docx]

**Supplementary Figures**


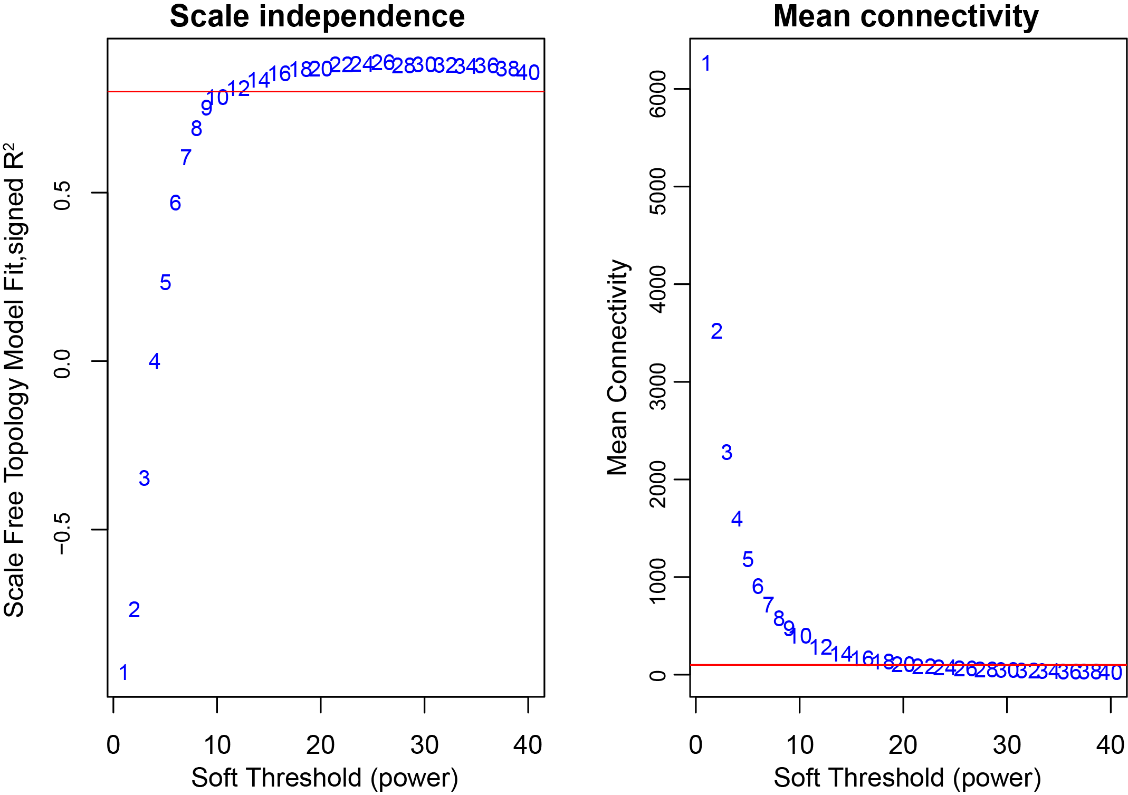


**Supplementary Figure** 1 | Identification of the soft-thresholding power

Note: the red line is where the value is 0.8 or 100 in Scale independence and Mean connectivity, respectively.


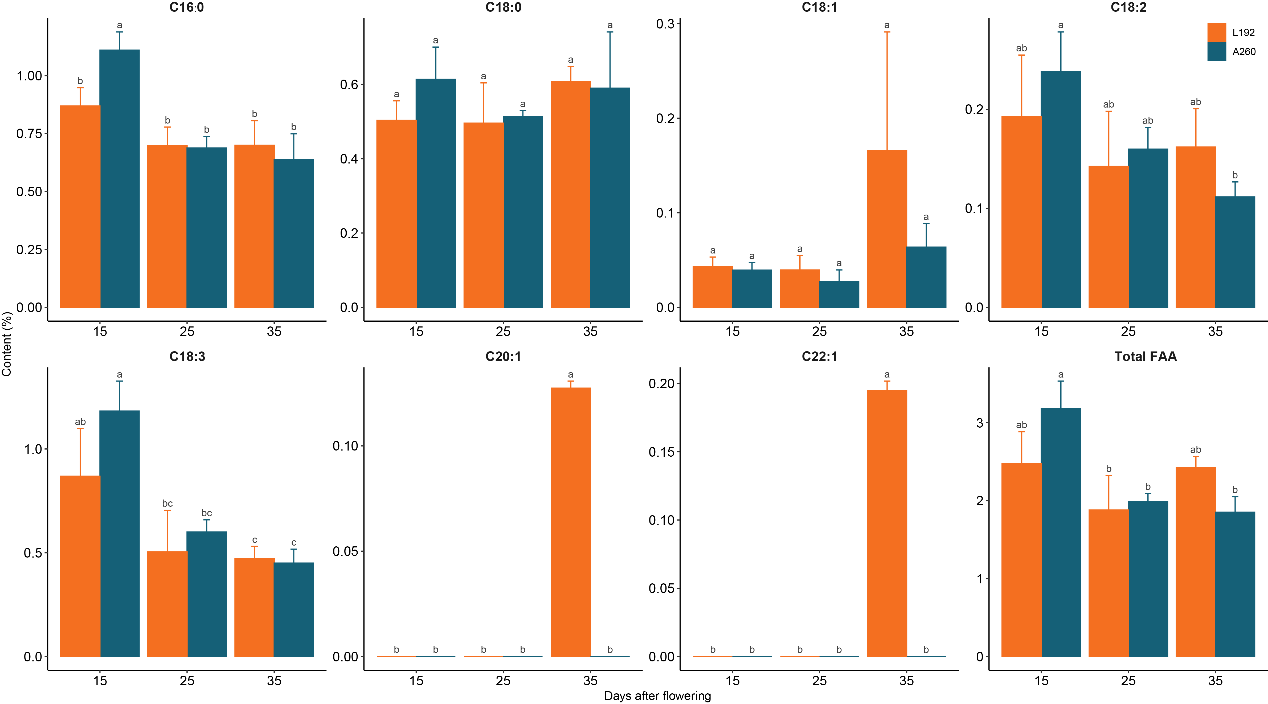


**Supplementary Figure 2** **|** Changes in fatty acid content during the development of silique walls

Note: The bars represent the means of three biological replicates, and error bars are standard deviations. Different letters marked on bars indicate significant differences according to *t* test analyses (p < 0.05) among groups.


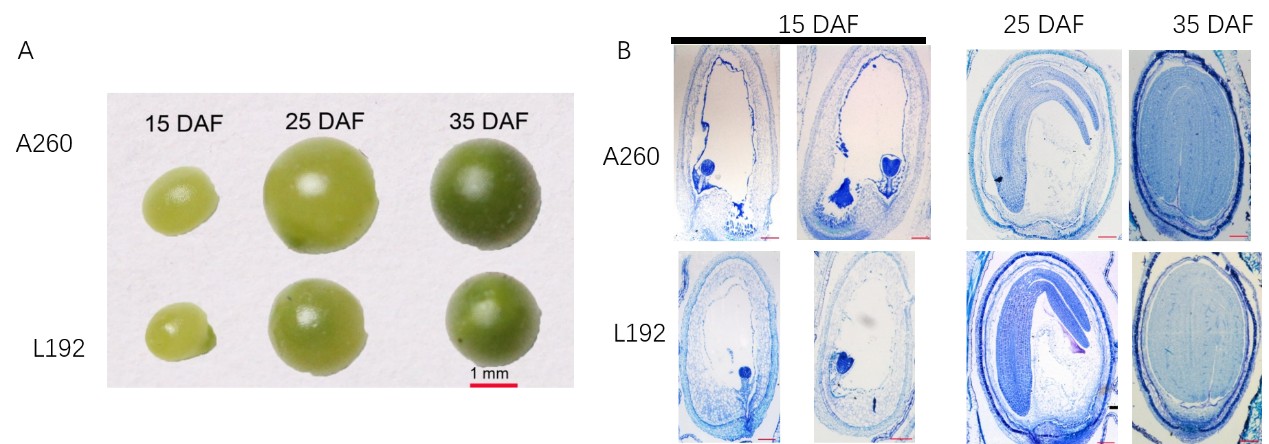


**Supplementary Figure 3 |** Development of seeds and histological structure of embryos

Note: A, fresh seed phenotype at 15, 25 and 35 DAF. B, Longitudinal sections of seeds at three stages. The bar is 1 mm.


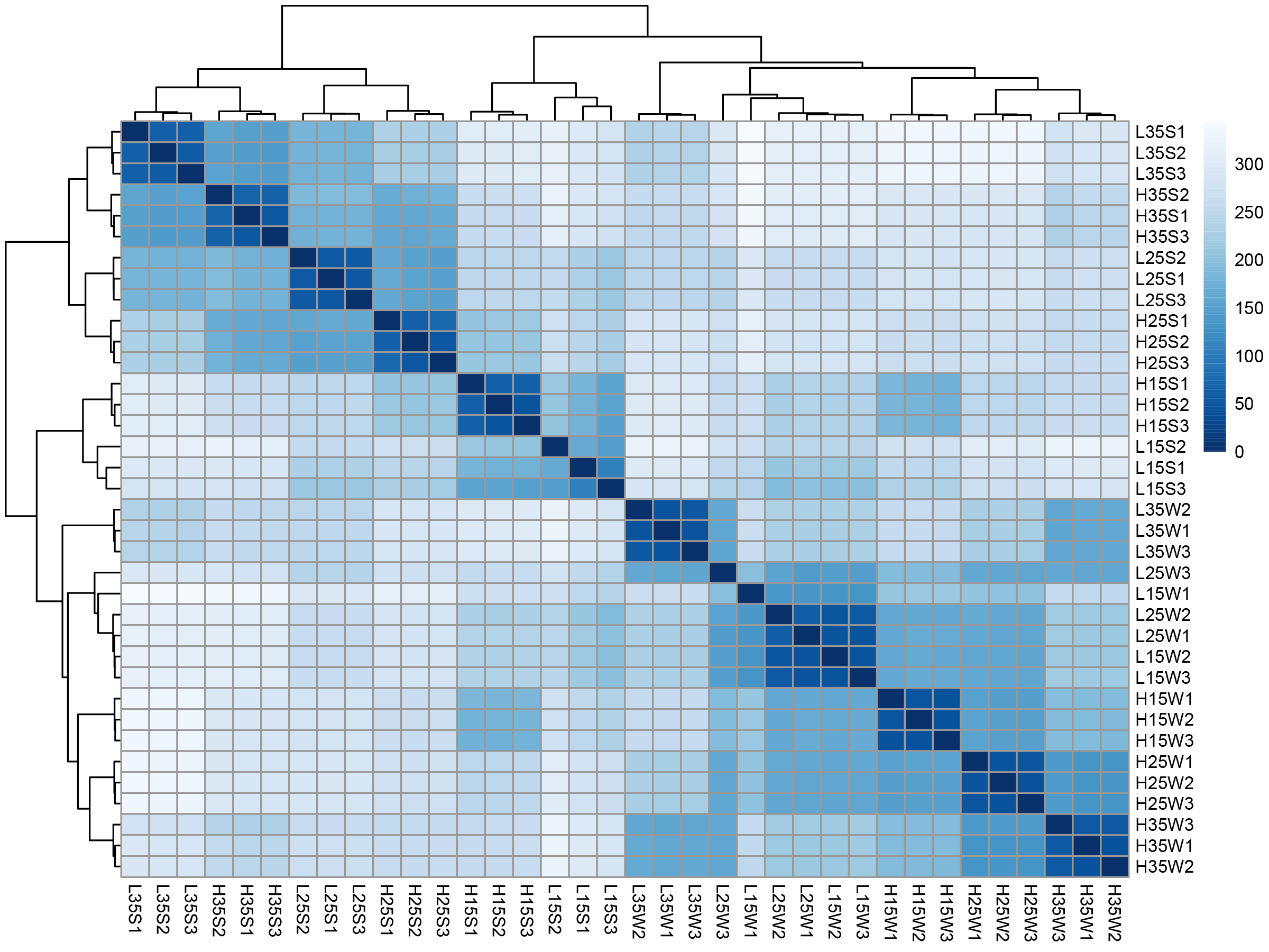


**Supplementary Figure 4 |** Cluster analysis of 36 samples

Note: H, samples of L192 (high oil content). L, samples of A260 (low oil content). S, seed. W, silique wall. 15, 25 and 35, 15, 25 and 35 days after flowering, respectively. The number at the end of the sample name was the code number of three biological replicates.


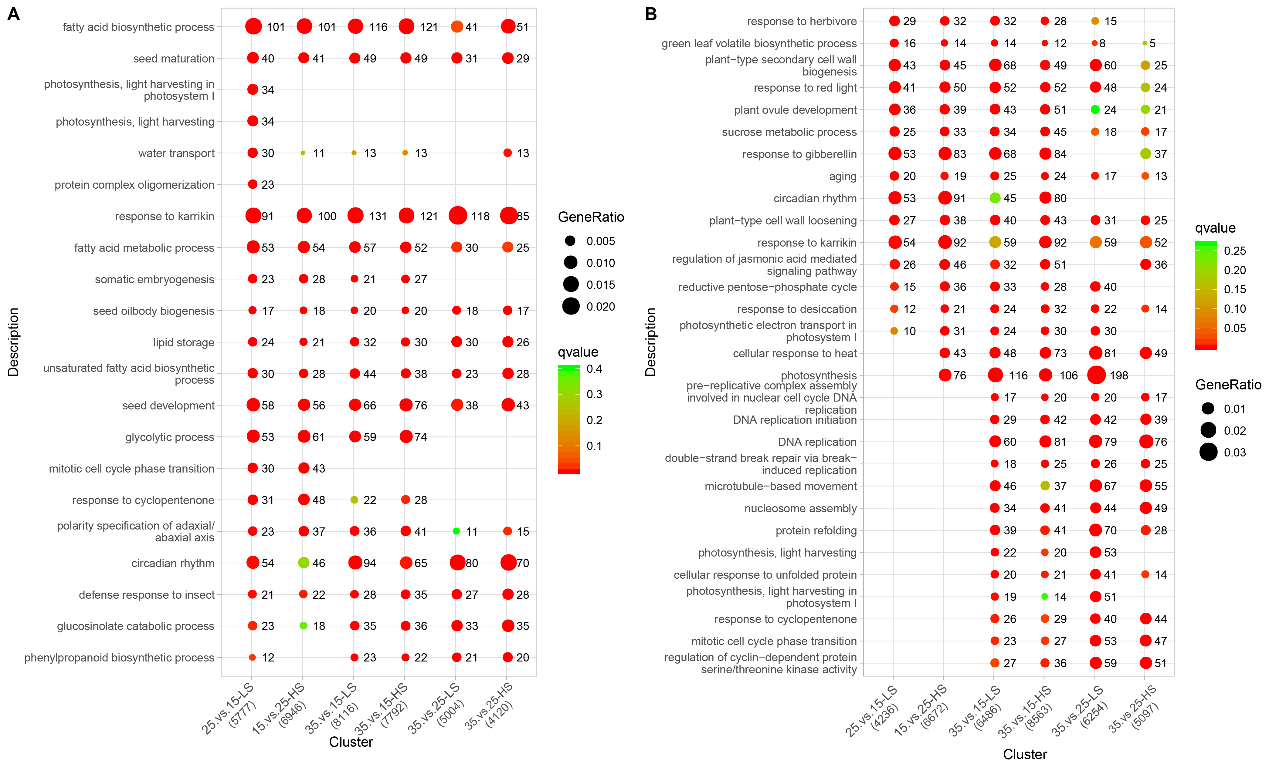


**Supplementary Figure 5** **|** GO enrichment analysis of genes during L192 and A260 seed development

Note: H, samples of L192 (high oil content). L, samples of A260 (low oil content). S, seed. 15, 25 and 35, 15, 25 and 35 days after flowering, respectively. Each group was a comparison of different development stage of seeds, for example, 25.vs.15-HS represented comparison between seeds of 25 DAF versus 15 DAF of L192. The numbers next to the each comparison was the total number of DEGs.


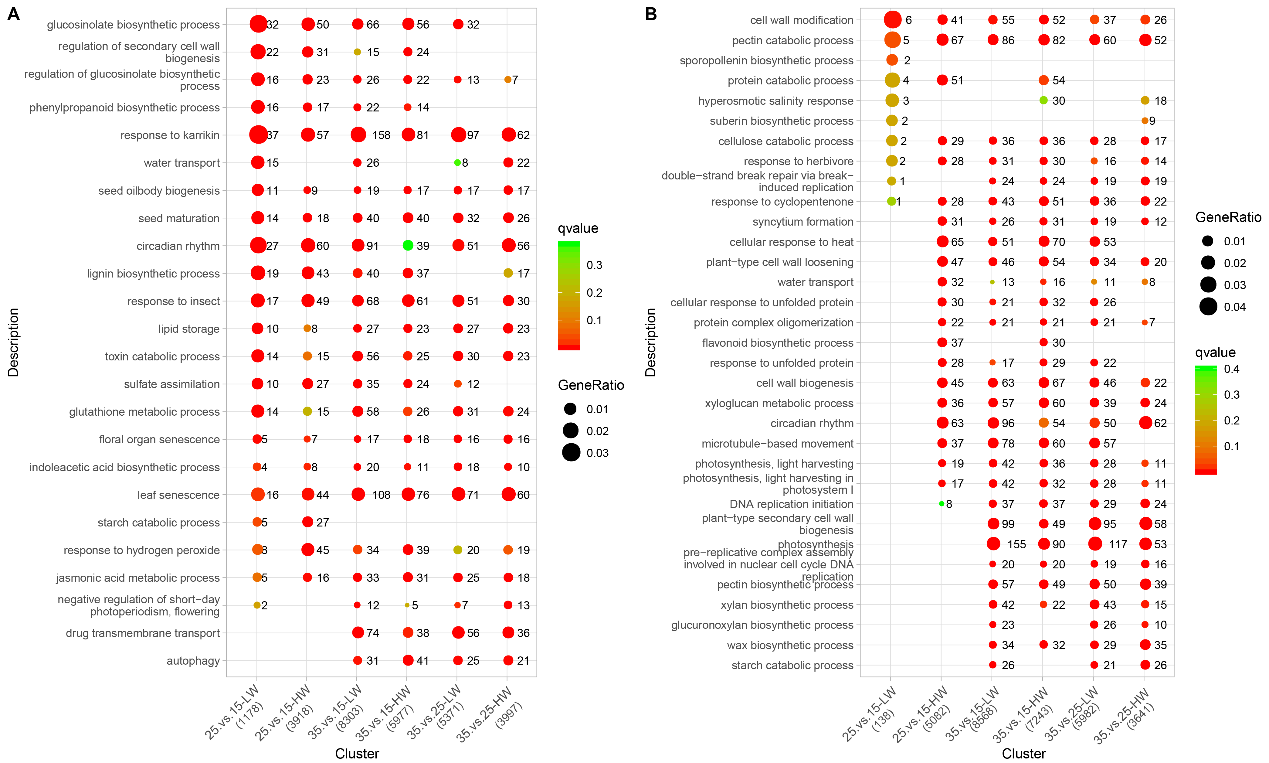


**Supplementary Figure 6 |** GO enrichment analysis of genes during L192 and A260 silique wall development

Note: H, samples of L192 (high oil content). L, samples of A260 (low oil content). W, silique wall. 15, 25 and 35, 15, 25 and 35 days after flowering, respectively. Each group was a comparison of different development stage of silique walls, for example, 25.vs.15-HW represented comparison between silique walls of 25 DAF versus 15 DAF of L192. The numbers next to the each comparison was the total number of DEGs.


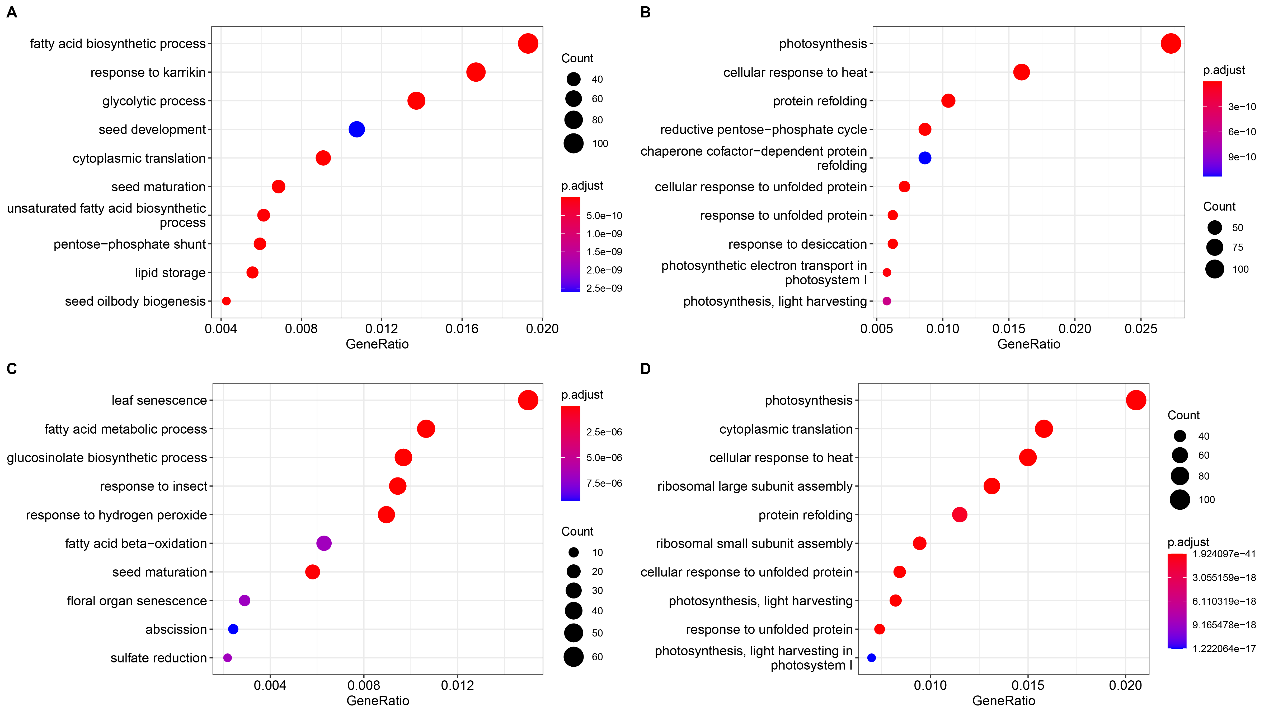


**Supplementary Figure 7** **|** GO enrichment analysis of genes over time

Note: A and B, biological processes enhanced or inhibited over time-course were enriched in the seed development, respectively. C and D, biological processes enhanced or inhibited over time-course were enriched in the silique wall development, respectively.


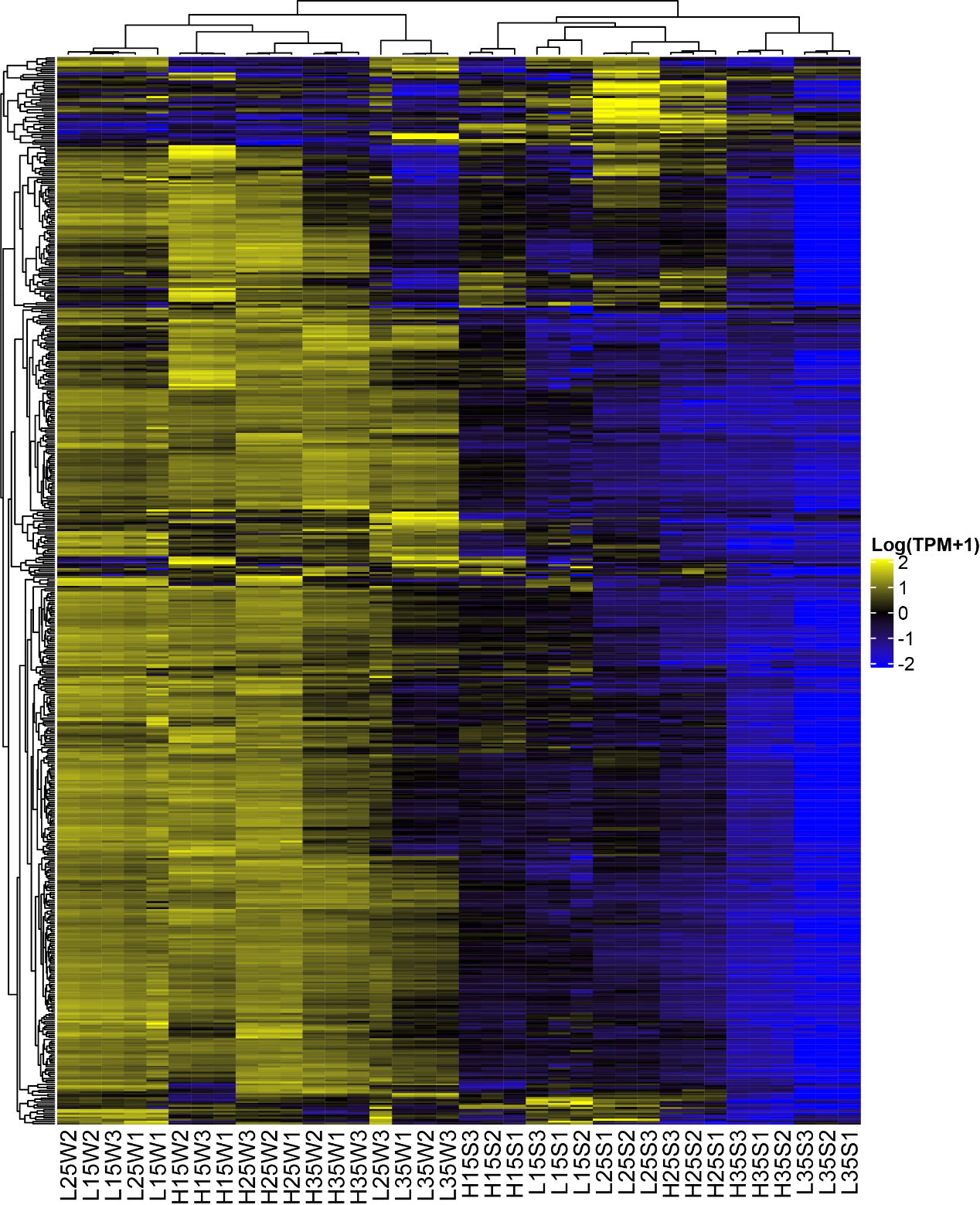


**Supplementary Figure 8** | Cluster analysis of genes related to photosynthesis among 36 samples

Note: H, samples of L192 (high oil content). L, samples of A260 (low oil content). S, seed. W, silique wall. 15, 25 and 35, 15, 25 and 35 days after flowering, respectively. The number at the end of the sample name was the code number of three biological replicates.
